# Overexpression of OASL upregulates TET1 to induce aberrant activation of CD4^+^ T cells in systemic sclerosis via IRF1 signaling

**DOI:** 10.1186/s13075-022-02741-w

**Published:** 2022-02-19

**Authors:** Zhuotong Zeng, Yaoyao Wang, Yangfan Xiao, Jie Zheng, Ruizhen Liu, Xinglan He, Jiangfan Yu, Bingsi Tang, Xiangning Qiu, Rui Tang, Yaqian Shi, Rong Xiao

**Affiliations:** 1 Department of Dermatology, The Second Xiangya Hospital, Central South University, Changsha, 410011 Hunan China; 2 Hunan Key Laboratory of Medical Epigenetics, The Second Xiangya Hospital, Central South University, Changsha, 410011 Hunan China; 3 Department of Dermatology, Sir Run Run Shaw Hospital, Zhejiang University School of Medicine, Hangzhou, 310016 Zhejian China; 4 Department of Anesthesiology, The Second Xiangya Hospital, Central South University, Changsha, China; 5 Clinical Nursing Teaching and Research Section, The Second Xiangya Hospital, Central South University, Changsha, China; 6 School of Life Science and Technology, Tongji University, Shanghai, 200092 China; 7 Dermatology Hospital of Fuzhou, Fuzhou, 350000 Fujian China; 8 Department of Rheumatology, Second Xiangya Hospital, Central South University, Changsha, 410000 Hunan China

**Keywords:** Systemic sclerosis, DNA methylation, CD4^+^T cell, OASL, IFN signaling

## Abstract

**Background:**

Systemic sclerosis (SSc), an autoimmune disease with unknown etiology and pathogenesis, is characterized by abnormal autoimmunity, vascular dysfunction, and progressive fibrosis of skin and organs. Studies have shown that a key factor in the pathogenesis of SSc is aberrant activation of CD4^+^ T cells. Our previous studies have shown that a global hypomethylation state of CD4^+^ T cells is closely related to aberrant activation. However, the exact mechanism of hypomethylation in CD4+T cells is not yet clear.

**Methods:**

Illumina HiSeq 2500 Platform was used to screen differentially expressed genes and explore the role of OASL, TET1, and IRF1 in the abnormal activation of CD4+T cells in SSc. Finally, double luciferase reporter gene experiments were used to analyze the interaction between IRF1 and TET1.

**Results:**

OASL overexpression could upregulate TET1 to increase the hydroxymethylation levels of CD4+ T cells and induce high expression of functional proteins (CD40L and CD70), thus promoting CD4+T cell aberrant activation. Moreover, OASL upregulated TET1 via IRF1 signaling activation, and a double luciferase reporter gene experiment revealed that IRF1 can bind to the TET1 promoter region to regulate its expression.

**Conclusions:**

OASL participates in the regulation of abnormal hypomethylation of CD4+ T cells in SSc, which implies a pivotal role for IFN signaling in the pathogenesis of SSc. Regulating DNA methylation and IFN signaling may serve as therapeutic treatments in SSc.

**Supplementary Information:**

The online version contains supplementary material available at 10.1186/s13075-022-02741-w.

## Introduction

Systemic sclerosis (SSc) is an autoimmune disease characterized by autoimmune abnormalities, microangiopathy, and progressive fibrosis of the skin and various organs [1–3]. However, the pathogenesis of SSc has not been fully clarified. Studies have shown that CD4^+^ T cell aberrant activation is a key factor in the pathogenesis of SSc [4, 5]. The CD4^+^ T cells of SSc patients are present in an autoreactive condition, which not only stimulates B cells to produce more autoantibodies, but it also releases various cytokines, resulting in inflammation, microvascular damage, and fibrosis [2, 6, 7].

DNA methylation is a form of DNA chemical modification that can change gene expression without changing the DNA sequence [8, 9]. DNA hydroxymethylation is a new type of DNA methylation that converts methyl- to hydroxymethylcytosines by ten-eleven translocation methylcytosine dioxygenase (TET) proteins, subsequently achieving demethylation [10, 11]. Recently, DNA hydroxymethylation has been found to play a pivotal role in tumorigenesis, inflammation, and autoimmune disease [11–15]. Our previous studies have also found that global hypomethylation of CD4+T cells is related to their abnormal expression of functional proteins such as CD40L and CD70, thus contributing to aberrant activation of CD4+T cells in SSc [16–20]. However, the exact mechanism of DNA hypomethylation of CD4+T cells in SSc patients has not been clarified yet.

Type I interferon (IFN) signaling is a key regulator of the innate immune system that modulates immune cell differentiation, proliferation, and cytokine production [21, 22]. 2′-5′-oligoadenylate synthetase like (OASL) is a member of IFN signaling, often as a key antiviral factor induced by IFNs [23]. Recent studies have found that OASL may participate in the pathogenesis of the autoimmune disease [24]. SSc is also defined as an IFN signature disease, which refers to increased expression and activation of IFN-regulated genes, such as MCP1/CCL2, OAS2, IFI30, and STAT1 [25]. It is well known that CD4^+^ T cells from SSc patients share a substantial number of hyper-expressed genes compared to healthy controls, most of which are induced by IFN signaling [26] and are involved in the pathogenesis of SSc. It remains to be answered whether IFN signaling regulates DNA hypomethylation and thus contributes to the overexpression of immune-related genes such as CD70, CD11a, and CD40L in CD4+T cells of SSc.

In this study, we examined the different gene expression profiles of CD4^+^ T cells in SSc patients and healthy controls and found that the expression level of OASL was abnormally increased in CD4^+^ T cells of SSc patients. Furthermore, we found that the overexpression of OASL upregulated TET1, leading to pathological hydroxymethylation of CD4^+^ T in SSc, increasing the expression of functional proteins CD40L and CD70. In addition, OASL induced IRF1 binding to the promoter region of TET1 to upregulate its expression. These findings revealed a pivotal role for IFN signaling in regulating DNA methylation, which helps clarify the pathogenesis of SSc, and identified OASL as a potential therapeutic target in SSc.

## Materials and methods

### Samples and patients

SSc patients from the Department of Dermatology, Second Xiangya Hospital were diagnosed as having SSc for one year according to the 2013 ACR/EULAR classification criteria [27]. These patients had never received immunosuppressive treatment which was summarized in Table 1. The healthy controls were obtained from healthy volunteers without any autoimmune diseases. The study was approved by the Institutional Review Board of Central South University and all of the subjects provided informed consent.

**Table 1:** The clinical features of patients

Patients	Gender	Age	Antibody	Disease duration	Therapy	mRSS	IDF	Digestive tract involvement
1	Male	49	ATA	2 years	None	36	1	1
2	Female	35	ATA	4 years	None	40	1	1
3	Female	48	ATA	1 year	None	27	1	0
4	Female	49	ATA	1 year	None	35	1	0
5	Female	56	ATA	3 years	None	41	0	1
6	Female	36	ATA	9 months	None	38	1	0
7	Female	60	ATA	2 years	None	22	1	0
8	Female	59	ATA	5 months	None	25	1	0
9	Female	36	ATA	1 year	None	20	0	1
10	Male	27	ATA	6 months	None	29	0	0
11	Male	61	ATA	3 years	None	30	1	1
12	Female	58	ATA	1 year	None	28	1	0
13	Female	55	ATA	5 years	None	32	1	1
14	Male	25	ATA^1^	8 months	None	15	0	0
15	Male	60	ATA	4 years	None	31	0	1

*ATA* anti-scl70 antibody positive, *Therapy* immunosuppressive therapy or glucocorticoid therapy, *mRSS* modified Rodnan skin score, *IDF* idiopathic pulmonary fibrosis; 1: yes; 0: No

No.1–5 patients were selected for RNA-seq

### CD4^+^T cell isolation

We collect 60ml/people peripheral blood for CD4^+^T cells isolation. CD4^+^ T cells were isolated by positive selection using CD4 immunomagnetic beads described in our previous study.

### RT-qPCR

The methods of Total RNA extraction, cDNA synthesis, and RT-qPCR were described in our previous study. The sequences of primers are presented in Table 2. β-actin was used as the endogenous control, and the relative expression level of each mRNA was calculated using the 2^-ΔCT^ method.

**Table 2:** PCR primer sequences

Gene	Forward primer sequences (5′-3′)	Reverse primer sequences (5′-3′)
OASL	CCACTTGACAGTGGAGCAGA	GGGATGGTCTCCAGCAGATA
Tet1	CCGAATCAAGCGGAAGAATA	CCTGGAGATGCCTCTTTCAC
IRF1	GTCCAGCCCACCTCTGTCTA	CAGGTCCTGCTTGCCTAGAG
IRF2	ACCTCCAGGTCACCATCAAA	CCTGGGTGATATCCGATGTT
PAX5	CCCTACAGCCACCCTCAGTA	TCATGGGCTCTCTGGCTATC
FOXP3	ACACGCATGTTTGCCTTCTT	TGTTCGTCCATCCTCCTTTC
STAT4	CAACAGAGCCACATTCTCCA	CCTTTCTTGGTGCGTCAGAG
CD40L	TAGCCAGCCTCTGCCTAAAG	CTTGGCTTGGATCAGTCACA
CD70	TGGTACACATCCAGGTGACG	AAGTGTCCCAGTGAGGTTGG

### Western blot

A total of 2 × 10^6^ cells were used to extract protein samples with a mixture of 100μlRIPA buffer (Beyotime, # P0013B) and 2 μl PMSF (Sigma, #329-98-6). The concentrations of protein were measured using a BCA Protein Assay Kit (Thermo Fisher Scientific, #NCI3225CH). The total protein (50 μg/sample) added in the loading buffer was boiled at 100 °C for 5 min, separated in a 10% gel at 80 V for 90 min, transferred to a membrane at 4°C using 300 mA for 85 min, and then incubated with QuickBlock™ Western (Beyotime, #P0252) for 10min. The membranes were probed with antihuman TET1 antibody (Abcam #ab191698), anti-OASL (Abcam #ab229136), anti-IRF1 (Abcam #ab191032), and anti-β-actin (Abcam #ab8227)) overnight at 4 °C. Horseradish peroxidase (HRP)-conjugated anti-rabbit IgG antibody (Abcam#) served as a secondary antibody

### The level of hydroxymethylation of CD4^+^ T cell detection

MethylFlashTM Hydroxymethylated DNA Qμantification Kit (Colorimetric) Whole Genomic DNA Hydroxymethylation Kit was used. The protocol is performed by manuscripts. The results were read at 425 nm.

### Flow cytometric analysis

Isolated CD4^+^ T cell suspensions (3 × 10^6^ cells) were stained with antihuman monoclonal antibodies CD4-FITC and CD25-APC (BD, USA), a tube of CD4^+^T cell (1 × 10^6^ cells) were incubated FITC-CD40L and FITC-CD70 (20μl) as described previously.

### siRNA treatment

OASL, TET1, PAX5, SATA4, IRF1, IRF2, and FOXP3 siRNA is provided by Guangzhou RiboBio. The siRNA (30nM) is added in CD4^+^T cell for 48h.

### Plasmid transfection

6 × 10^6^ cells/well planted in the 6-well, the OASL, TET1 Flag plasmid (4μg/well), and IRF1 (5μg/well) plasmid were transfected for 48h.

### Dual-luciferase reporter gene analysis

HEK293T cells were plated in a 12-well plate (6 × 10^5^ cells/well) and cultured in DMEM medium overnight, then co-transfected with either plz1 (0 to +600 bp deletion), plz2 (0 to+100 bp deletion), plz3 (− 500 to − 300 deletion), plz4 (0 to +100 and − 500 to − 300 deletion), or plz5 (− 300 to 0bp deletion) mutIRF1 according to the manufacturer’s instructions. Dual-luciferase reporter assays were performed 48 h post-transfection using the dual-luciferase reporter assay system (Promega, #E1910, USA) according to the manufacturer’s instructions.

### RNA seq

RNA were extracted from CD4+ T cells of samples using the TRIzol reagent. RNA-Seq was carried out on the Illumina HiSeq 2500 according to the manufacturer’s protocol.

### DATA analysis

DESseq2 was used to perform data processing and differential gene selection on the compared count expression profiles. The BH method was used to adjust *p*-values, and adjusted *p* values of ≤ 0.05 and absolute values of log2 FoldChange ≥ 1 were used as thresholds to select differentially expressed genes. DESeq2 for GSEA analysis was used, and the selected annotation datasets were KEGG, REACTOME, and GO.BP with *p* values ≤ 0.05 as thresholds to select up- and downregulated pathways.

### Statistical analysis

The data are presented as the mean ± SD and performed in Graphpad6.0. The comparison between each groups were made by using Student’s *t* test, or the nonparametric test. *P* < 0.05 is defined as significance (*P < 0.05, **P < 0.01,***P < 0.001,****P < 0.0001).

## Results

### Differentially expressed genes in peripheral blood CD4^+^ T cells between SSc patients and healthy controls

**Fig. 1:**
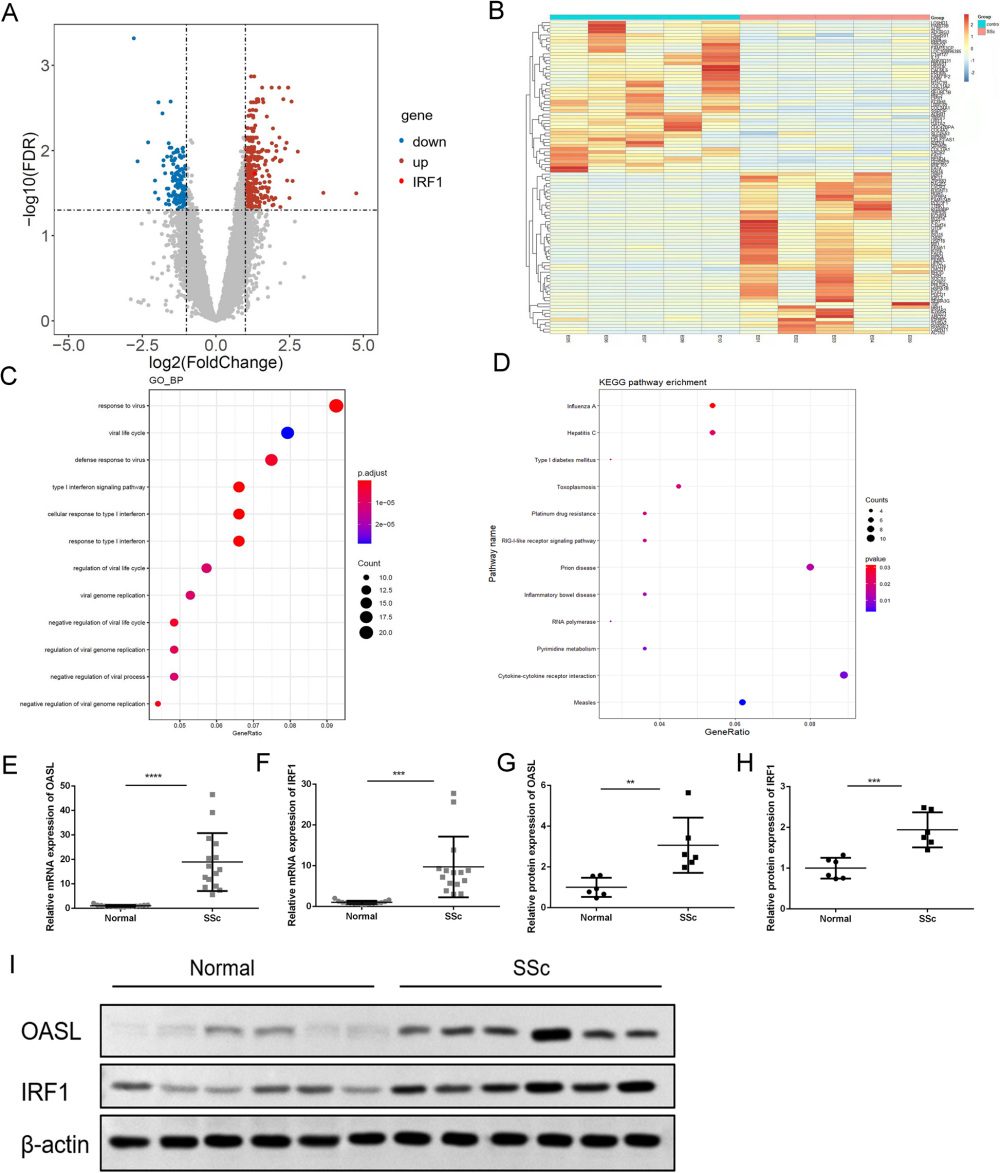
Identification of differentially expressed genes between systemic sclerosis and normal. **a** Volcano plot of the differential gene expression analysis. *X*-axis: fold change difference (log 2 scale); *y*-axis: BH-adjusted *p* values for each probe (-log10 scale). **b** Hot map for differential gene expression analysis. The vertical dotted lines represent an absolute cutoff value of 1.5-fold change. The horizontal dotted line represents the significant cutoff of *p* < 0.05. **c** GO enrichment analysis of DEGs; **d** KEGG pathway analysis of DGEs. The horizontal axes shows -log10 transformed *P* value and *p* < 0.05 is considered significant. **e**, **f** The mRNA expression level of OASL and IRF1 in CD4^+^T cells of SSc patients (*n*=15). **g**–**i** The protein expression level of OASL and IRF1 in CD4+T cells Of SSc patients (*n*=6)

To elucidate the function and molecular changes of CD4^+^ T cells in the pathogenesis of SSc, we first isolated CD4^+^ T cells from five SSc patients and five age- and sex-matched healthy donors (HDs), and used the Illumina HiSeq 2500 Platform to screen differentially expressed genes (DEGs). After screening and a comparison based on the fold change and false discovery rate (FDR), we use volcano graphs to visualize the expression levels of differential genes between SSc patients and HDs (Fig. 1a). There were a total of 260 DEGs between SSc patients and healthy controls. Of these, 175 genes were upregulated and 85 downregulated (Fig. 1b). To identify the specific pathways that were altered, we performed GO analysis and observed that these genes were mainly enriched in the type I interferon signaling pathway and in response to viruses, as well as in the regulation of lymphocyte activation. KEGG pathway analysis highlighted measles, cytokine-cytokine receptor interaction, RIG-I-like receptor signaling, and pyrimidine metabolism (Fig. 1c, d). Among these genes, we identified two IFN-related genes (OASL and IRF1) that were upregulated in SSc patients and may be involved in the pathogenesis of SSc. We expanded the sample size to verify the expression levels of OASL and IRF1 in CD4^+^ T cells. The results showed that the mRNA expression levels of OASL and IRF1 were indeed increased in CD4^+^T cells of SSc patients (Fig. 1e, f), as were the protein levels (Fig. 1g–i).

### Overexpression of OASL upregulates CD40L and CD70 expression levels via increasing DNA hydroxymethylation in CD4^+^ T cells of SSc patients

**Fig. 2:**
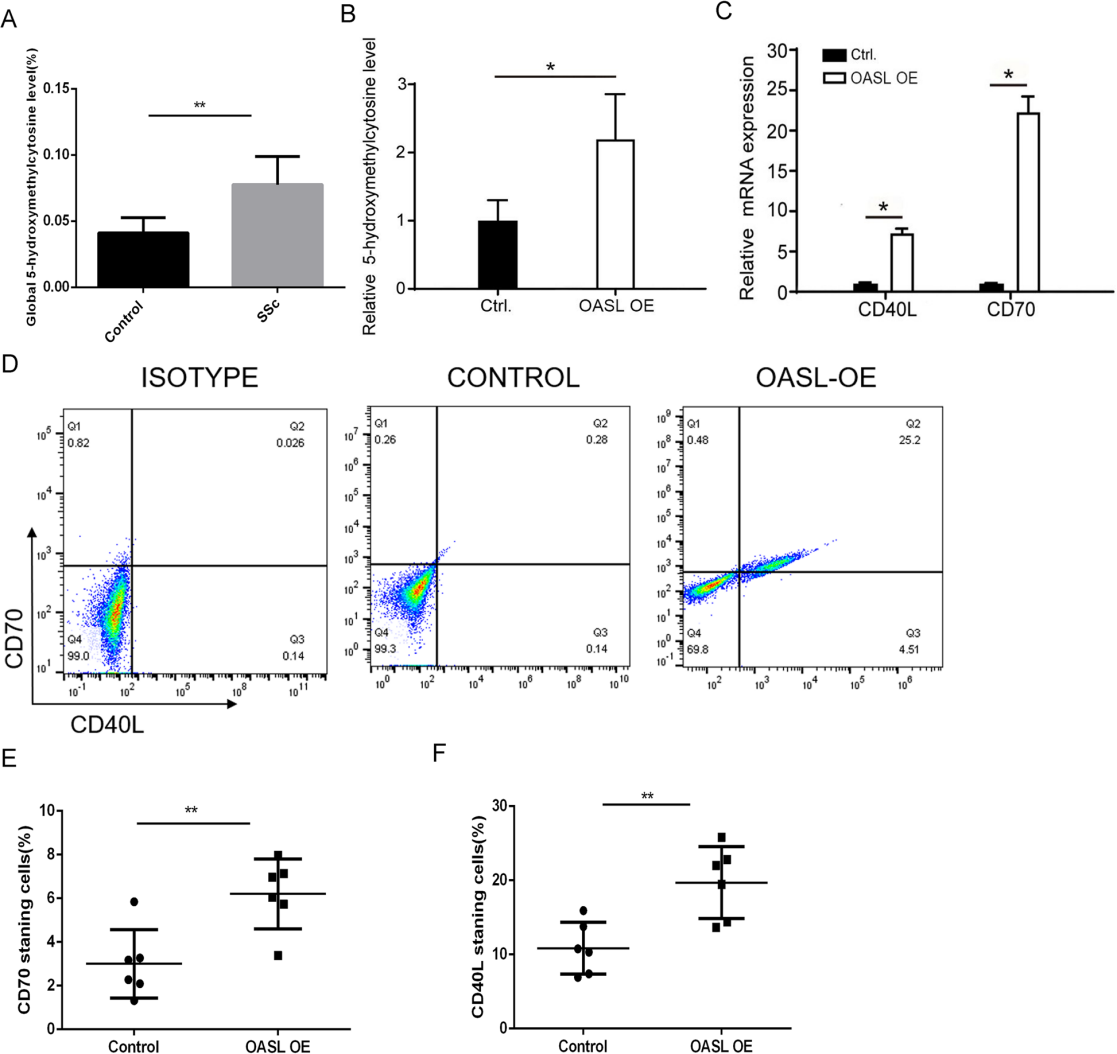
Overexpression of TET1 upregulated the CD40L and CD70 expression level via increasing of DNA hydroxymethylation in CD4^+^ T cells of SSc patients. **a** Global 5-hydroxymethylcytosine level of CD4+T cells in SSc patients (*n*=10). **b** The hydroxymethylation level of CD4^+^ T cells after OASL-Flag recombinant plasmid treatment in normal CD4^+^T cells. **c** The mRNA expression level of CD40L and CD70 in CD4^+^T cells of SSc. **d**–**f** The percentage of CD40L and CD70 staining cells (*n*=6)

It has recently been found that OASL may act as a biomarker or predictor of therapeutic responses in autoimmune disease [24, 28–31]. In previous studies, we found that hypomethylation of CD40L and CD70 in their promoter regions contributes to their overexpression in CD4^+^ T cells of SSc patients and induces an aberrant autoimmune response [16, 17]. We first confirmed that the overall level of hydroxymethylation significantly increased in CD4^+^T cells of SSc patients (Fig. 2a). We next sought to identify whether overexpression of OASL is involved in this pathological process. We transfected normal CD4^+^ T cells with OASL-Flag recombinant plasmids to confirm whether OASL overexpression can mimic the abnormal activation state in CD4^+^ T cells of SSc. The results showed that the overall level of hydroxymethylation significantly increased in normal CD4^+^ T cells after OASL plasmid transfection (Fig. 2b). Also, the expression levels of CD40L and CD70 significantly increased (Fig. 2c–e).

### TET1 overexpression upregulates CD40L and CD70 expression levels via increased DNA hydroxymethylation in CD4^+^ T cells of SSc patients

**Fig. 3:**
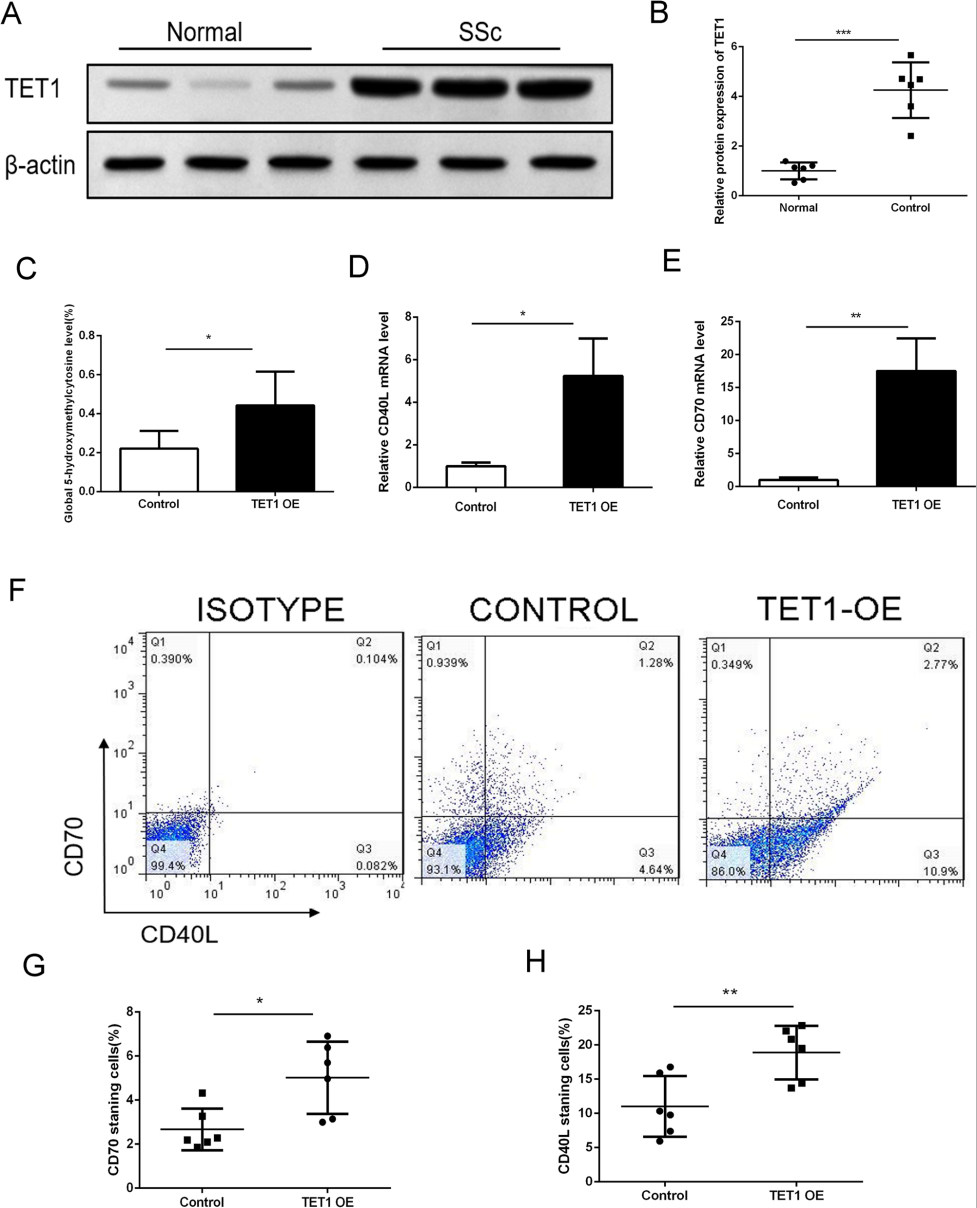
Overexpression of OASL upregulated the CD40L and CD70 expression level via increasing of DNA hydroxymethylation in CD4^+^ T cells of SSc patients. **a**, **b** The expression level of Tet1 in CD4^+^T cells of SSc. **c** The hydroxymethylation level of CD4^+^ T cells after TET1-Flag recombinant plasmid treatment in normal CD4^+^T cells. **d**, **e** The mRNA expression level of CD40L and CD70 in CD4^+^T cells after OASL-Flag recombinant plasmid treatment in normal CD4^+^T cells. **f**–**h** The percentage of CD40L and CD70 staining cells after OASL-Flag recombinant plasmid treatment in normal CD4^+^T cells (*n*=6)

TET1 is a member of the TET family that can oxidize 5-methylcytosine (5mC) to 5-hydroxymethylcytosine (5hmC), thereby driving the completion of DNA demethylation. Recently, Tet protein has been considered to be the main mediator of the increase in global 5hmC levels and the present hypomethylation state in cells, which plays an important role in the process of tumorigenesis, inflammation, and autoimmune diseases [32, 33]. We hypothesized that the DNA demethylase TET1 also participates in hypomethylation in CD4^+^ T cells of SSc patients. We first identified its expression levels in SSc patients and normal healthy controls (Fig. 3a, b) and found that TET1 is enhanced in CD4^+^ T cells of SSc. We further explored the exact role of TET1 in the activation of CD4^+^ T cells. Our findings demonstrated that overexpression of TET1 with a plasmid can upregulate the hydroxymethylation level (Fig. 3c) in addition to the CD40L and CD70 expression levels in normal CD4^+^ T cells (Fig. 3d–h). These results indicate that TET1 may participate in the pathogenesis of SSc via increasing hydroxymethylation levels in CD4^+^ T cells.

### OASL upregulates the expression level of TET1 in CD4^+^ T cells of SSc patients

**Fig. 4:**
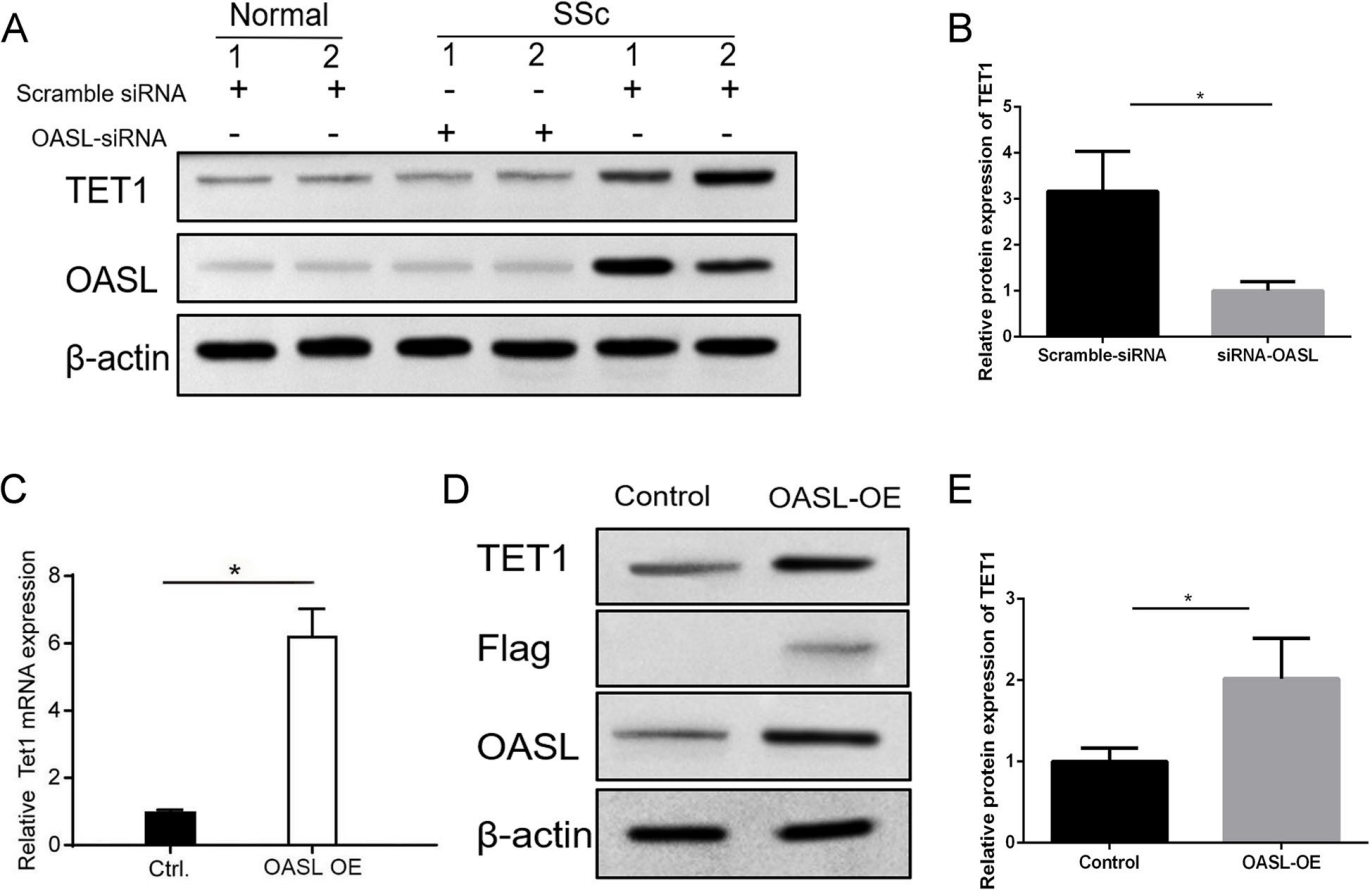
Overexpression of OASL upregulated TET1. **a**, **b** Expression level of Tet1 decreased after OASL-siRNA treated in CD4^+^ T cells of SSc patients. **c**–**e** The expression level of Tet1 increased after OASL-Flag recombinant plasmid treatment in normal CD4^+^T cells (*n*=3)

Since we found that the overexpression of OASL and TET1 commonly increase the hydroxymethylation level of CD4^+^ T cells, we wondered whether OASL could regulate the expression of TET1. We first silenced OASL expression to observe whether it influenced the expression of TET1. The results showed that the expression level of TET1 decreased after OASL-siRNA treatment of CD4^+^ T cells of SSc patients (Fig. 4a, b), while the overexpression of OASL increased TET1 expression levels in normal CD4^+^ T cells (Fig. 4c–e).

### OASL upregulates expression level of TET1 via IRF1 signaling

**Fig. 5:**
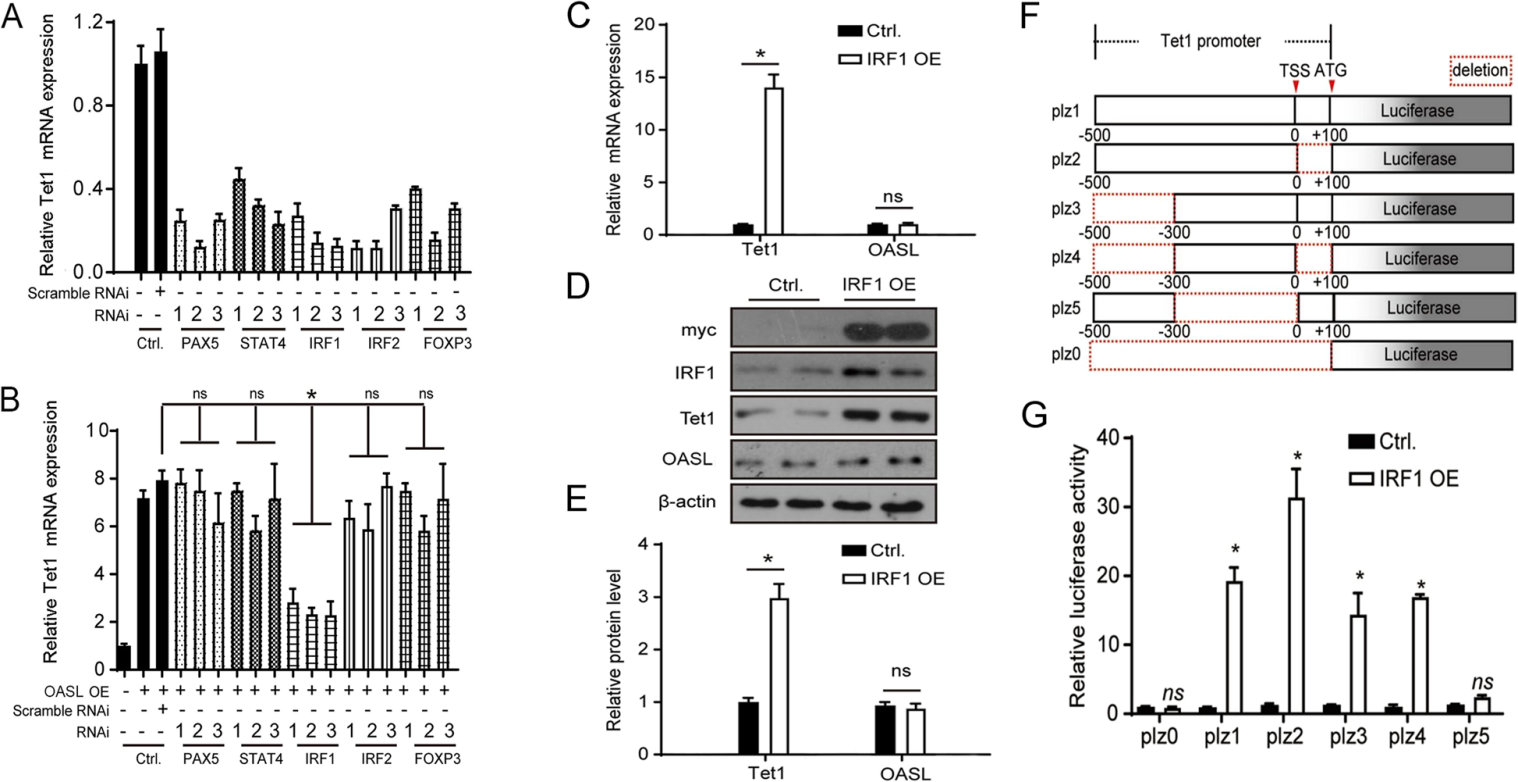
OASL upregulated TET1 via stimulating IRF1 signaling. **a**, **b** The mRNA expression level of Tet1 after transcription factors silenced in normal CD4^+^T cells transfected with OASL-Flag recombinant plasmid. **c**–**e** The expression level of TET1 in normal CD4^+^T cells after transfected with IRF1-Flag recombinant plasmid. **f** Conservation analysis of IRF1 binding sites in TET1 promoter. **g** Dual-luciferase reporter assay detected relative luciferase activities after cotransfection of five truncated IRF1 promoters with pcDNA3.1 vector in HEK293T cells (*n*=3)

To explore the exact mechanism of how OASL regulates TET1, we selected several possible immune-related transcription factors (PAX5, STAT4, IRF1, IRF2, and FOXP3) by analyzing the 5′-UTR region of Tet1. We then detected the expression levels of TET1 after interfering with these transcription factors in normal CD4^+^ T cells transfected with an OASL-Flag recombinant plasmid. The results showed that the ability of OASL to upregulate TET1 expression was significantly weakened only after IRF1 was silenced in normal CD4^+^ T cells transfected with the OASL-Flag recombinant plasmid (Fig. 5a, b). These results were consistent with our expression profile chip in which IRF1 is upregulated in CD4^+^ T cells of SSc. Then, to explore the exact role of IRF1 in the mechanism by which OASL regulates TET1 expression, we transfected an IRF1-myc recombinant plasmid in normal human CD4^+^ T cells. The results showed that the expression levels of Tet1 mRNA and protein were significantly increased in normal human CD4^+^ T cells transfected with the IRF1-myc recombinant plasmid; however, the expression of OASL was not influenced (Fig. 5c–e). We used the transcription start site as the 0 position and constructed a full-length 600 bp deletion in the 5’-UTR region of Tet1 (plz1), a 0 to +100 deletion (plz2), a − 500 to − 300 deletion (plz3), a 0 to +100 deletion combined with a − 500 to − 300 segment deletion (plz4), and a − 300 to 0 deletion (plz5). These plasmids and IRF1-myc plasmid were co-transformed into HEK293 cells. The dual-luciferase reporter assay shows that plz1-4 could obviously generate fluorescent signals, while the plz5 plasmid lacking the −300 to 0 fragment did not generate clear fluorescent signals (Fig. 5f, g). These results indicate that IRF1 could upregulate TET1 by binding to the promoter region of TET1.

## Discussion

Our previous study confirmed that CD4^+^ T cells of SSc patients have global hypomethylation [20], and we further found that DNA hypomethylation in the promoter regions of immune-related genes, such as CD40L and CD70, contribute to their hyperexpression and promote abnormal activation of CD4^+^ T cells [17, 19, 20]. However, the exact mechanism by which CD4^+^ T cells acquire pathological hypomethylation is not fully known. TET1 is a member of the TET family, which consists of enzymes that oxidize 5mC to 5hmC, eventually leading to DNA demethylation. Lu et al. found that the overexpression of TET2 and TET3 promotes global DNA hydroxymethylation, thus inducing the overexpression of many immune-related genes in CD4^+^ T cells of SLE patients [34]. Hattori et al. have shown that TET1, but not TET2 or TET3, is upregulated in skin dermal fibroblasts of SSc patients and contributes to global DNA hypomethylation [35]. These data indicate that TET proteins participate in the pathogenesis of autoimmune diseases through DNA methylation [36–38]. We wondered whether the DNA demethylase TET1 plays a pathogenic role in the aberrant activation of CD4^+^ T cells in SSc. Our study supports the above findings and provides further molecular details. In the present research, we found that upregulation of TET1 enhances global DNA hydroxymethylation and increases the expression of immune-related genes CD40L and CD70, eventually promoting the aberrant activation of CD4^+^ T cells in SSc patients.

On this basis, we further explored factors upstream of TET1 to identify DEGs that might regulate it in CD4^+^ T cells in SSc patients. We identified OASL as significantly upregulated in CD4^+^ T cells of SSc patients by using expression profiling chips. OASL is a 2′-5′ oligoadenylate synthase (OAS) family member that is a key antiviral factor induced by IFNs [23, 39, 40]. OASL is essential to fight viral infections in the innate and adaptive immune responses, usually via IFN signaling [23], and has recently been shown to be a biomarker or predictor of therapeutic responses in autoimmune diseases. Some studies have suggested that OASL and OAS2 are upregulated in PBMCs and skin tissue of SSc patients [41], and OAS family genes are also increased in CD31^+^/CD102^+^ lung microvascular endothelial cells from SSc patients with end-stage interstitial lung disease [42]. However, these studies have not clarified the mechanism by which OASL is involved in the pathogenesis of SSc. We revealed that OASL promoted global DNA hydoxymethylation levels, enhanced CD40L and CD70 expression levels, and induced the activation of CD4^+^ T cells. Moreover, we also found that OASL overexpression could upregulate TET1 in normal CD4^+^ T cells and that OASL silencing inhibited TET1 expression in CD4^+^ T cells of SSc patients, which suggests that OASL may play a pathogenic role in the activation of CD4+T cells in SSc through DNA hydroxymethylation mediated by TET1.

In addition to OASL, most of the DEGs screened out through our expression profiling chips in CD4^+^T cells were enriched in the I-IFN pathway (see Fig. 1c). Interferon regulatory factors (IRFs) are transcription regulators that contain a conserved helix-turn-helix DNA binding motif that recognizes IFN-stimulated response elements (ISRE) in the promoter regions of other genes that participate in many cellular processes, including proliferation, response to tumors and viruses, and immune regulation [43, 44]. IRF1 is an IRF that is a master regulator of type I IFN signaling, which could be regulated by OASL through the RIG-1 pathway [23, 45, 46]. Recently, a GWAS study identified IRF1 as a susceptibility gene in SSc [47]. Our findings also demonstrated that the expression level of IRF1 is enhanced in CD4^+^ T cells of SSc. Interestingly, there is a predicted IRF1 binding site in the TET1 promoter region. To determine whether OASL upregulates TET1 expression via IRF1, we further explored the functional relationship between TET1 and IRF1. Our data showed that overexpression of IRF1 could significantly stimulate the expression of TET1 in normal CD4^+^ T cells. In addition, a high expression level of IRF1 was verified in CD4^+^ T cells of SSc patients. Moreover, IRF1 silencing reversed the overexpression of TET1, but not OASL, in CD4^+^ T cells of SSc patients. Importantly, the dual-luciferase reporter assay confirmed that IRF1 can bind to the promoter region of TET1. These observations demonstrate the importance of OASL-IRF1 induced abnormal immune responses in SSc. To the best of our knowledge, this is the first study to identify the regulatory role of IFN signaling in CD4^+^ T cells with aberrant activation via DNA methylation in SSc.

## Conclusions

In this study, we demonstrated that OASL, IRF1, and the hypomethylation-related gene TET1 were significantly upregulated in CD4^+^ T cells of SSc patients. OASL upregulates TET1 to increase the global DNA hydroxymethylation level, enhancing CD40L and CD70 expression levels and inducing aberrant activation of CD4^+^ T cells. Importantly, we identified IRF1 as a key transcriptional regulator between OASL and TET1. We found that OASL could upregulate TET1 expression via IRF1 signaling activation, and IRF1 knockdown ameliorated the TET1 expression level, but did not influence OASL. These results demonstrate that OASL mediates global hydoxymethylation and induces abnormal activation of CD4^+^ T cells in SSc, which is thought to upregulate TET1 expression through IRF1 signaling activation.

Taken together, these data reveal a pathogenic role for OASL and its possible mechanism in the abnormal activation of CD4^+^T cells. We conclude that type I IFN signaling and its downstream pathway factors OASL and IRF1 are important mediators in the pathogenesis of SSc by regulating DNA methylation. OASL and IRF1 may therefore serve as potential therapeutic targets in SSc.

## Supplementary Information

**Additional file 1.**

## Data Availability

The analyzed data sets generated during the present study are available from the corresponding author on reasonable request.

## Abbreviations

SSc: Systemic sclerosis
TET: Ten-eleven translocation methylcytosine dioxygenase
IFN: Type I interferon
OASL: 2′-5′-oligoadenylate synthetase like
HRP: Horseradish peroxidase
DEGs: Differentially expressed genes
FDR: False discovery rate
5mC: 5-methylcytosine
5hmC: 5-hydroxymethylcytosine
IRFs: Interferon regulatory factors
